# Getting Married

**Published:** 1876-01

**Authors:** 


GETTING MARRIED.

Into this gulf of perdition will they arrive at no remote day who arrange to go to boarding as soon as married, instead of going to housekeeping. A more unprofitable mistake cannot possibly be made by the newly married; it is more mischievious in winter than in summer, for in warm weather the young wife can walk on the street, or drive in the Park, or visit among her friends, but in the winter they are cooped up in one room, and spend hours at the window-pane, gazing listlessly out opon the street; afraid of the cold, yet detesting the confinement, while a great part of the time, the husband being at his business, the mind of the young wife is the prey of a thousand disquietudes; at one time she thinks her friends are slighting her; at another, she becomes envious of others who seem to be able to dress more elegantly than herself, and she begins to make unfavorable comparisons to herself and to her husband, as to their worldly condition, and she becomes moody, or petulant, and complains, and the husband wakes up to a new discovery, and as unwelcome as it is new—that his wife is not happy; not happy in him, not happy in herself, not happy in her social position. But suppose these same persons had begun life in the old fashioned way; had taken a small house and had busied themselves first in arranging whatever parental affection had bestowed, and then had set about deciding what they could afford to take from their own store of money and expend it for the most necessary articles of furniture, and as to the things they wanted besides, things not indispensable, yet desirable, laying plans together how, by economizing, first in one direction and then in another, they might lay aside enough to procure what they had set their minds upon; and when this was accomplished, then to have another aim. No man can doubt that in going to housekeeping in such a manner, the prospect of a happy and thrifty married life is unmistakably more promising than in the health and heart destroying practice of living the first twelve months of married life in hotels and boarding houses. Very few young wives are safe in any public associations. The papers abound in cases of infidelity to marital engagements by lately marri ed girls being thrown into the society of men of leisure, their husbands being engaged in their business pursuits. The mere dandy, loafer, or gentleman of leisure has every opportunity of taste, and dress, and address above that of the mere man of business, to turn away a woman's heart from her husband by the mere fact of causing her at first, without implicating himself, to draw unfavorable comparisons against her husband, or to personal tidiness. One of the most splendid weddings that had ever taken place then, in New York, resulted in a seperation, and a broken heart, and a premature grave within a few years, by the wife's continual twitting her husband, when they were walking on Broadway or the Avenue, about his want of taste in the selection of his apparel; —"why don't you bow as Mr. Blank does?"—"His gloves are in perfect taste; yours are such as a countryman would select."

These things grew upon her, while they alienated him, and living as they did at the finest hotel in the city, with uncounted gold at their command, and nothing to compel their attention away from trifling things, their minds dwelt upon them, magnified them, allowed them to see nothing else, with the result of a seperation under circumstances which were infinitely worse than death, for then the grave would have closed over every sorrow, whereas life was spared, with its long years of agony.

### THE MEN WON'T PROPOSE.

Because they are afraid of the enormous expenses of housekeeping. It requires a little fortune, now, to buy a house, and every article of furniture costs about three times as much as it did ten years ago. Young men of spirit, and they are the only ones worth having now, begin to calculate the cost of wedlock. When they see the extravagant lengths to which our daughters go in their dress; when they look at the splendid mansions in which their fathers live, their minds begin to run in this channel; "She is a charming girl, in fact, too good for me, but to place such a trusting creature in a condition inferior to the one in which she now finds herself, would be dishonorable, and I must forego the happiness of marrying her, even were she willing, until I have obtained the means of placing her in a social position worthy of her;" and while he is bending his energies to bring about this end, years creep on; opinions have changed, views of life have altered; the affections have become chilled and the mind hardened with its attritions with men, preferences have been diverted, and in too many cases an old bachelor and an old maid occupy the places which otherwise might have been the abode of a happy family and a delightful association.

Every body ought to get married who can boast of three things, First, A sound body. Second, A sound mind. Third, A good trade; this as to men; and as to women, they should possess good health, tidiness and industry. With these, any young couple can get as rich as they ought to be, or as rich as is necessary to an enjoyable life, if they will only go to housekeeping a little below their ability.

The young should have the courage to live within their means; to have more pride in the consciousness that they have a little spare money at home, than in living in a style which keeps them all the time cramped in maintaining. Better to live in one room with all the furniture your own than occupy a whole house with scarcely a chair or table paid for.

### WORKING TOGETHER.

It is productive of a great deal of domestic enjoyment to have a man and wife working to the same end, having a common object in view, whether it be to save up money enough to buy a new chair, or a neighbor's adjoining farm. It is thus that they grow into the feeling that they are one, that their interests are united, and they soon begin to work into each other's hands; the wife seeks to make the husband's task easier, knowing that it enables him to do more for her, and that the common object will be the sooner attained; the husband seeing this, reciprocates, and loves the more, day by day, until they become one in aim, and feelings, and sentiments, and a love more abiding as the years grow old, is the happy result.

### HELP FATHER.

"I do not mind being sick so much, nor the pain connected with it, but father is getting old, and he needs my help at the store."

This was the sentiment of a young man the other day, who was suffering a temporary disablement. There was something beautiful in it.

Every night about dark, a small boy with a light ladder and a box of matches, may be seen running along the street. He stops at each lamp post, lights the gas, and then runs to another; he does not loiter; does not stop to talk with other boys, or to gaze at the splendid equipages which are yet returning from the Park along the Avenue. He is the son of the man whose business it is to light the city lamps. No hired boy could possibly be procured who would perform the duty with such alacrity and fidelity, too; for not long ago he passed a lamp-post a rod or two and on looking back, he discovered it was burning too high. He at once ran back and adjusted it properly. What if it did burn too high; it was nothing to him, nor to the city, nor to his father; but suppose one of the Directors of the gas companies had chanced to see that wasting light, his father might have lost his place. A lesson may be learned here which would add a million fold to family happiness, and it is this—it increases the affections of families to have the sons and daughters brought up to assist their parents in household duties, or in the means of family support; there grows up a community of interest, each feels that he is helping the others, and the affections naturally go out towards those who help us. If a mother has no other use of her daughter, she should require her to assist in all domestic matters; and sons should be early learned to proffer their services to their parents, even if it be to save a few steps in walking, or to make the accomplishment of any work in hand less laborious; in short, children should be so educated that it shall be to them a duty, a pleasure and a happiness to help their parents, and to do it with alacrity, with a spontaneous promptitude which never gives time to be asked. But most parents will plead guilty to the charge, that instead of requiring their children to help them, and wait upon them in many little ways, they too often prefer to wait on or help themselves.

There is a feeling, which if expressed in words would read thus—"The child may be engaged in something else of its own, or may not like to do it, and I can almost as easily do it myself; at all events I won't divert him from his own objects." Many a mother has made a bed in weariness, or cooked a dinner, or swept a room, or washed a garment, or set up half the night in sewing, patching or knitting, when her daughters could have shared the labor and made it easy to both; but that daughter's dress might have been soiled, or she had a visitor, or was going to take a walk, or was finishing a story.—Mothers, remember this one important truth—the most self-sacrificing parents are oftenest brought to an old age of grief by the misconduct of both sons and daughters.

Selfish children, those who are not allowed to bear the famly burdens soon begin to feel that it is not their place and that their parents ought to devote themselves to their comfort and happiness, and the sentiment expresses itself in words sometimes, "It is no more than they ought to do." "Other children's parents do it, and why not mine?" Nothing is more true than that those children who take the least interest in household matters, or in the business of the family, are the most selfish, the most ungrateful, and the least unlikely to grow up useful members of society, while those who are always ready to help their parents, to share their labors, will grow up more and more affectionate, more and more worthy of parental love, and are very certain in after life to be found the praise of the community in which they live.

THE UNDERSTANDINGS

of the physical man has a great deal to do with health and life. Many a man and woman owe an untimely death to damp feet in winter-time. This is very generally admitted, and many methods have been proposed to prevent it. In wet weather, or when the snow is melting, the India rubber shoe is the most perfect article offered; some prejudice has been excited against them, more than anything else from the unwise use of them. They may be hurtful to some, but it does not follow that they are generally so.

No one can be comfortable with cold, damp feet, and the very instant it is noticed, the person should begin to walk, or remove both stockings and hold the bare feet to the fire until they are perfectly dried and feel comfortably warm. India rubber overshoes should be worn only when the person is walking; as soon as the walk is ended they should be removed. They certainly ought not to remain on the feet ten minutes if the person is standing still in the house after a walk. If a person is in and out many times in a day, it is better to have a pair of old shoes covered with good India Rubber to remain on permanently, to be slipped on and off together; another pair of shoes and slippers, to be worn in their stead while indoors; but unless this other pair is always kept warm when not in use, the removal of a shoe and slipping on a cold one, will give a bad cold or a troublesome rheumatic affection in a very short time. The great mass of business men cannot make these changes, and to them the old fashioned soled shoe or boot is best.

Various expedients have been devised to keep the dampness from the soles of the feet. Some advise that a piece of sail cloth or other woven material, should be cut in the shape of the sole, dipped in melted pitch or tar, and when cooled, placed between the layers of the shoe's sole and well sewed. If this is carefully done it is simply impossible for any dampness to penetrate to the soles of the feet by simply walking on damp ground; but in walking in wet grass or the slosh of snow deep enough to reach the upper leather, this device is no protection.

Another means of rendering soles of shoes impervious to dampness, and to prevent their squeaking, is to set them in melted tallow deep enough to merely cover the soles, and let them remain a week; if it is in a mixture of equal parts of beeswax and tallow it is still the better.

A gentleman avers that from six years of experience and trial, the soles of shoes are not only made water-proof but will last three times as long if a coat of gum copal varnish is applied to the soles and repeated as it dries, until the pores of the leather are filled, and the surface shines like polished mahogany.

The soles of shoes may be made impervious to water by rubbing the following mixture into the leather, until it is thoroughly saturated—One pint of boiled linseed oil; half a pound of mutton suet; six ounces of pure beeswax; four ounces of rosin. Melt these over a slow fire, stirring well, and when the shoes are new, warm them and the mixture also, and use.

Or put a pound each of rosin and tallow in a pot on the fire and when melted and mixed, apply while hot, with a painter's brush, to both soles and upper leather. If it is desired that the boots should take a polish immediately, dissolve an ounce of beeswax in a teaspoonful each of turpentine and lamp black a day or two after the boots have been treated with the rosin and tallow, rub over them this wax and turpentine, away from the fire. Thus the exterior will have a coat of wax alone, and will have a bright polish. Tallow and grease become rancid and rot the stitching, and the leather also; while the rosin mixture preserves both.

One pint of linseed oil, a quarter of a pint of turpentine or camphor, a quarter of a pound of beeswax and a quarter of a pound of Burgundy pitch. Melt together with a gentle heat; warm it when it is to be used, and rub it into the leather before the fire, or in the sun.

Or, melt together beeswax and mutton suet, half and half, and rub it in where the stitches are.

Gutta Percha soles are preferred by some. They may be attached thus: dry the old sole, roughen it well with a rasp, and rub on with the finger a thin, warm solution of gutta percha; dry it, hold it to the fire, and then rub on a coat of a thicker solution. Take the gutta percha sole, soften it in hot water, wipe it, and hold both sole and shoe to the fire until warm; lay the sole on gradually, beginning at the toe. In half an hour, pare it neatly with a knife.

But it must be remembered that if you make the upper leather of a shoe water-tight, it is rendered measureably air-tight, and this occasions dampness on the inside, creating ill odors and coldness, while any kind of oily substance must not only rot the material but cause a noisome smell.

To those who are forehanded, and have leisure, it is advised to purchase the shoes to be worn in winter six months before hand, and wear them a little at a time in warm weather; thus they become hardened before winter sets in, and this hardening increases their durability. But before they are once worn in the wet, the soles should be held to the fire until they are well warmed; then warm a little tar in a tin cup, and apply it with a swab to the bottom of the shoe, but not hot enough to burn the leather, then let it be well dried in before the fire. This will never work out while warming the feet; but this tar should be applied the first of each month until May, if the boots are worn much in the wet. This tar penetrates the sole to the eighth of an inch, and renders it almost as hard as horn. Grease of any kind will soften the leather and make it porous. Without this tar application, the first wetting of the soles will contract them and making them fit not so well, sometimes making them too small altogether.

If shoes are heated before the fire, they get hard and wear out very much sooner than if allowed to dry gradually in the upper part of the kitchen or family room, farthest from the fire, or on a shelf, or hung on a nail.

VARNISH FOR SHOES.

It is a bad plan to grease the upper leather of shoes for the purpose of keeping them soft; it rots the leather and admits dampness more readily. It is better to make a varnish thus;

Put half a pound of gum shellac, broken up in small pieces, in a quart bottle or jug, cover it with alcohol, cork it tight and put it on a shelf in a warm place; shake it well several times a day, then add a piece of gum camphor as large as a hen's egg; shake it well, and in a few hours shake it again and add one ounce of lamp black; if the alcohol is good, it will all be dissolved in three days; then shake and use. If it gets too thick, add alcohol—pour out two or three teaspoonfuls in a saucer and apply it with a small paint brush. If the materials were all good, it will dry in about five minutes, and will be removed only by wearing it off, giving a gloss almost equal to patent leather.

The advantage of this preperation above others is, it does not strike into the leather and make it hard, but remains on the surface and yet excludes the water almost perfectly.

This same preperation is admirable for harness, and does not soil when touched, as lamp black preperations do.

### COLD FEET WHILE TRAVELING.

If boots are treated as above, and just before going out of doors the stockings are removed, and both feet and stockings are well dried before the fire, the feet will feel comfortably warm for several hours; it is the moisture or steam about the feet which often makes them feel cold by the out-door air condensing them. No one should travel in winter with tight-fitting shoes; they arrest the circulation: this induces coldness, causing a general feeling of discomfort all over the body, even making the mind fretful and irritable. A woolen stocking will alone keep the feet warmer than the same stockings and a pair of tight boots besides. If a person has a good circulation, the feet will get warm of themselves if the tight boots are removed. No one can go to bed with cold feet without doing themselves a positive injury; and it is always best in winter-time, even if the feet do not feel cold, at bed-time to draw off the stockings and hold the feet to the fire or stove, rubbing them meanwhile with the hand, until they are perfectly dry and comfortably warm in every part; it is a pleasant operation of itself, and ought not to be dispensed with for a single night from October to May; it is one of the best anodynes; it allows a person to [illegible] asleep in five minutes, who, with cold feet, would have remained awake for half an hour or more, and even then the sleep will be unrefreshing and dreamy.

### BURNING FEET.

If the soles are dry and hot at bed time, rub patiently into each one of them, with the hand, half a teaspoonful of sweet oil night after night, until the difficulty is removed.

Some persons always have cold feet on getting into bed; a robust person may remedy this in time by dipping both feet at a time in cold water just deep enough to cover the toes; let them remain in until thirty are counted, wipe dry, hold to the fire, and jump into bed.

Feeble persons and invalids should pursue a different course. Put both feet in hot water half leg deep; add hot water from time to time for fifteen minutes, so that the water shall be hotter when they are taken out then when they are put in, then dip them in cold water as before, while you count ten, wipe warm, and get into bed.

As cold feet induces a number of diseases, aggravates others, and delays the cure of all, it is worth all the trouble one can take if thereby, even in the course of months, the delightful condition can be brought about wherein the feet are in such a natural and healthful state, that the mind is never attracted towards them unpleasantly.

